# Effects of unilateral vs. bilateral resistance training interventions on measures of strength, jump, linear and change of direction speed: a systematic review and meta-analysis

**DOI:** 10.5114/biolsport.2022.107024

**Published:** 2021-07-03

**Authors:** Kai-Fang Liao, George P. Nassis, Chris Bishop, Wei Yang, Chao Bian, Yong-Ming Li

**Affiliations:** 1 School of Physical Education and Sport Training, Shanghai University of Sport, Shanghai, China; 2 Department of Strength and Conditioning, Guangdong Vocational Institute of Sport, Guangzhou, China; 3 Physical Education Department, College of Education (CEDU), United Arab Emirates University, Al Ain, Abu Dhabi, United Arab Emirates; 4 Department of Sports Science and Clinical Biomechanics, Faculty of Health Sciences, SDU Sport and Health Sciences Cluster, University of Southern Denmark, Odense, Denmark; 5 School of Science and Technology, London Sport Institute, Middlesex University, London, UK; 6 China Institute of Sport Science, Beijing, China

**Keywords:** Exercise selection, Unilateral exercises, Bilateral exercises, Performance

## Abstract

Exercises can be categorized into either unilateral or bilateral movements. Despite the topic popularity, the answer to the question as to which (unilateral or bilateral) is superior for a certain athletic performance enhancement remains unclear. To compare the effect of unilateral and bilateral resistance training interventions on measures of athletic performance. Keywords related with *unilateral, bilateral and performance* were used to search in the Web of Science, PubMed databases, and Google Scholar and ResearchGate™ websites. 6365 articles were initially identified, 14 met the inclusion criteria and were included in the final analysis, with overall article quality being deemed *moderate*. The quantitative analysis comprised 392 subjects (aged: 16 to 26 years). Sub-group analysis showed that unilateral exercise resistance training resulted in a large effect in improving unilateral jump performance compared to bilateral training (ES = 0.89 [0.52, 1.26]). In contrast, bilateral exercise resistance training showed a small effect in improving bilateral strength compared to unilateral (ES = -0.43 [-0.71, -0.14]). Non-significant differences were found in improving unilateral strength (ES = 0.26 [-0.03, 0.55]), bilateral jump performance (ES = -0.04 [-0.31, 0.23]), change of direction (COD) (ES = 0.31 [-0.01, 0.63]) and speed (ES = -0.12 [-0.46, 0.21]) performance. Unilateral resistance training exercises should be chosen for improving unilateral jumping performance, and bilateral resistance training exercises should be chosen for improving bilateral strength performance.

## INTRODUCTION

Resistance training is one of the most widely used methods of enhancing athletic performance [1–3]. One of the challenging problems faced by practitioners is the issue of how to optimally choose the exercises to maximize training effects when prescribing resistance training programs. Typically, exercises in the weight room can be categorized into either unilateral or bilateral. A unilateral exercise is a weight bearing movement mainly or completely involving one limb (e.g. single leg squat, Bulgarian split squat and single leg jump), whereas, a bilateral exercise is a weight bearing movement executed evenly and simultaneously by both limbs (e.g. back squat, deadlift and countermovement jump). Traditionally, bilateral exercises are selected as the primary exercises for athletic development [4, 5] due to their effects on improving strength and power [6–9]. In contrast, unilateral exercises have been commonly considered to be ‘more supplementary’ for injury prevention [10]. However, many key sport-specific skills involved with the basic lower-body movements (e.g. running, changing direction, jumping, kicking) are executed completely or predominantly unilaterally. Under the specificity of training adaptation and to maximize the transfer of training [11], it could be argued that unilateral exercises similar to sport-specific skills, may be the best choice to improve athletic performance [10, 12], and prioritized as a key exercise in such training programs. But the answer to the question of which one (unilateral vs. bilateral) is better for athletic performance enhancement remains unclear.

Specificity of training exercises is crucial for transference of training-induced adaptations to the target performance [13]. Young [5] proposed that exercise should be as specific as possible to optimize the transfer of training. In addition, Bosch [14] proposed that intra-muscular and inter-muscular coordination, outer movement resemblance and energy production are the key factors to evaluate and predict the specificity of the training methods. A compelling body of empirical evidence also supports exercise type specificity with regards to the range of motions, velocities, postures, and patterns [13, 15–18]. Actually, except for the obvious mechanical differences, unilateral and bilateral exercises also differ in intra- and inter-muscular aspects such as interhemispheric mutual activation [19], postural stability [20], relationship of force and velocity [21], psychological state [22] and lumbar load [23]. Based on those distinctions, unilateral and bilateral resistance training are expected not to transfer equally.

According to the training principle of specificity, unilateral resistance training should improve unilateral performance measures better compared with bilateral resistance training, and vice versa. However, current findings are conflicting with respect to which is better for the improvement of measures of athletic performance [24–30]. Some studies support the notion that unilateral resistance training (e.g. Bulgarian split squat) improves unilateral strength more than bilateral exercise training (e.g. back squat) [24, 26], and some studies have found no differences between methods [25, 31]. There is also confusion about which method is superior for the improvement of change of direction (COD) speed performance, with some favoring unilateral training methods [32] and others favoring bilateral methods [26]. Furthermore, there is also conflicting evidence on the effects of these training methods on bilateral strength, jump and speed performance [24–26, 28, 31, 32]. Recently, a meta-analysis carried by Moran et al., [33] concluded that there was no difference between the effect of unilateral and bilateral resistance training on horizontal movement speed (ES = 0.17, p = 0.30), but noted that the effect size was pooled by 7 short sprints, 2 CODs, 1 five alternated leg bounding and 1 stair climb outcomes from 11 included studies. Given this review focused purely on the effects on horizontal speed, it is still unclear how both bilateral and unilateral exercises transfer to other key physical attributes, such as strength, jumping and COD speed. Therefore, the purpose of this systematic review and meta-analysis aimed to compare the effects of unilateral vs. bilateral resistance training on improving athletic performance. The hypothesis was that the effect of unilateral and bilateral resistance training would follow the principle of specificity. In other words, the unilateral resistance training would be better for improving the unilateral performance, and the bilateral resistance training would be better for improving the bilateral performance.

## MATERIALS AND METHODS

### Literature research

**FIG. 1:**
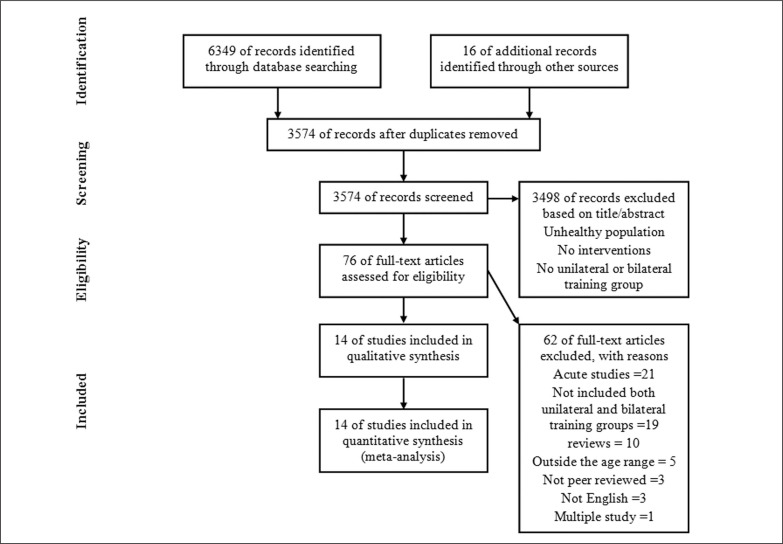
Flow chart illustrating the different phases of the search and study selection

This systematic review was conducted under the Preferred Reporting Items for Systematic Reviews and Meta-Analysis Protocol (PRISMA). One author searched the related articles from Web of Science (1980–2020) and PubMed (1949 to 2020). The following keywords inclusive of three main terms as *unilateral*, *bilateral* and *performance* were used and combined under Boolean’s language with the operators AND and OR. Term 1: *unilateral, split squat, single leg, one leg, step up, lunge, Bulgarian split squat*. Term 2: *bilateral, back squat, deadlift, double leg, two legs, hang clean, hang snatch.* Term 3: *performance, strength, resistance, speed, power, jump, agility, change of direction (COD), endurance*. If studies were not available in relevant electronic databases, then further searches were conducted in Google Scholar and Research Gate™ websites. Finally, additional studies were identified by checking the reference list of the selected articles. The final search date for literature was October 1, 2020 (Figure 1).

### Inclusion Criteria

Studies were eligible if they met the following criteria: (1) implemented both unilateral and bilateral resistance training interventions; (2) the duration of training was longer than 4 weeks; (3) the training intensity was moderate to heavy; (4) included healthy participants aged from 16 to 40 years old in both genders; (5) had measured athletic performance (Speed, strength, COD, power test etc.) before and after the training intervention; (6) presented full data (mean and SD) that allowed effect sizes to be calculated; (7) the manuscripts were written in English and were published in a peer-reviewed journal.

### Literature selection

The first author imported all records into Endnote software (X9.3.3) and deleted any duplicates. Then, the first and fourth authors checked the title and abstract separately to exclude any unrelated articles, with the remaining full texts screened against the inclusion criteria. There was no disagreement between the 2 authors on that aspect.

### Risk of bias assessment

The fourth and fifth authors independently assessed the selected studies. In case of disagreement on certain item scores, the item scores would be given after discussion. Considering the most risk of bias assessment scales such as Delphi scale, PEDro scale and Cochrane scale are designed for medical research, studies about training interventions usually get very low score under these methodological scales [34]. We preferred the scale (Table 1) modified by Brughelli et al [34] and Hooren et al. [35]. This scale is deemed more suitable for sport science research, and includes 10 items, with each item rated as: 0 = clearly no/not reported, 1 = maybe, and 2 = clearly yes. The articles were rated poor with a total score lower than 10, moderate with a score between 10 and 15, good with a score > 15, and excellent with a score equal to 20.

**TABLE 1:** Risk of bias assessment scale [34, 35]

No.	Items	Scores
1	Clear inclusion criteria	0–2
2	Clear description of the participants’ training experience	0–2
3	Random allocation of the participants to groups	0–2
4	Clearly defined intervention	0–2
5	Similarity test at baseline for all groups	0–2
6	Use of a control group that did not perform resistance training	0–2
7	Clearly defined outcome variables	0–2
8	Adequate familiarization period	0–2
9	Appropriate between-group statistical analysis	0–2
10	Point measures of variability	0–2
	Total	0–20

### Coding of the studies

The first author extracted the data from the selected literatures with a standard table. The code included: (1) participants: age, gender, identity and training experience; (2) interventions: frequency, duration, exercises, intensity and volume; (3) measurements and results: pre and post test outcome, with means and standard deviation.

### Statistical analysis

The review manager software (5.3) was used for the meta-analytic comparison if more than one outcome were evaluated for a certain kind athletic performance measure between unilateral and bilateral resistance training (e.g. Strength, speed), subgroup analyses were performed.

Chi^2^ and I^2^ were calculated to test the heterogeneity. For I^2^ values of 25, 50, and 75% represent low, medium, and high heterogeneity, respectively [36]. For Chi^2^ with large value and *p* < 0.1 show evidences of heterogeneity. If *p* > 0.1 and I^2^ < 50%, the fixed effects model was applied. Otherwise, the random effects model was applied and provoked further investigation through a subgroup analysis of moderator variables (Training experience, identity, gender, training frequency, training modalities, training weeks). In order to identify the presence of highly influential studies, a sensitivity analysis was executed by removing one study at a time. Studies were considered as influential if removal resulted in a change of heterogeneity (*p*) from significance (*p* < 0. 1) to non-significance (*p* > 0.1).

Standardized mean differences (SMD) was calculated with the following algorithm [37]:

[(M_post,unilateral_ – M_pre, unilateral_) – (M_post,bilateral_ – M_pre, bilateral_)/pooled SD_pre_]

This algorithm was selected as it has been recommended for effect size calculation of independent pre-/post- study designs in meta-analysis based on simulation results. The algorithm in speed and COD outcomes was adjusted as [M_pre_ – M_post_], of which the smaller values represent better results compared with other outcomes. SMD was used because the studies all evaluate the same outcome but test it in various methods such as maximum power of leg press (n.m) and 1RM of leg press (kg) in strength measures. The absolute values of effect sizes were rated with the following criterion given by Cohen [38]: < 0.2 as trivial, 0.2–0.49 as small, 0.5–0.79 as moderate, ≥ 0.8 as large. Values are reported with 95% confidence intervals to describe the range of the true effect. If the absolute value of aggregated effect and 95% confidence interval are above zero, effect size can be considered as clear evidence: A positive effect size indicated that the effect of unilateral exercises training was better for the improvement of athletic performance than bilateral exercise training, and a negative effect size indicates the opposite, i.e. bilateral better than unilateral.

Given that unilateral athletic performance tests in included studies are generally divided into the left and right leg separately, and 5 of included studies have used multiple measures of the same athletic performance such as 10 m sprint, 20 m sprint for speed performance (table 2), which may introduce statistical dependency into the meta-analytic data set and enlarge the type I error due to the same participants contributing to two or more effect sizes [39], we selected a single most relevant effect size to deal with the multiplicity according to a decision rule as following: 1) the most used test in included studies; 2) the right leg test; 3) in accordance with training practice: a) multiple joint movements > Single joint movements; b) Dynamic movements > isometric movements/isokinetic movements; 4) shorter links with the true athletic performance: Jumping performance: single jump > multiple jumps; change of direction: preference to the greater angle and shorter sprint distance.

**TABLE 2:** Multiplicity of included studies

Study	Athletic performance	Multiplicity of Outcome	Selected for meta-Analysis
Botton et al. [29]	Bilateral strength	Isokinetic knee extension	
		Knee extension 1RM	√
	Unilateral strength	Isokinetic knee extension	
		Knee extension 1RM	√

Ramirez-Campillo et al. [40]	Bilateral strength	Knee extension 1RM	√
		Knee flexion 1RM	
	Unilateral Jump	Countermovement jump	√
		Horizontal crossover triple jumps	
		Horizontal triple jumps	
		Squat jump	
	Bilateral Jump	Countermovement jump	√
		Squat jump	
		Horizontal jump	
		Horizontal triple jumps	

Stern et al. [25]	Unilateral Jump	Drop jump	
		Countermovement jump	√
		Horizontal jump	
	Bilateral Jump	Drop jump	
		Countermovement jump	√
	Speed	10 m	√
		30 m	

Gonzalo-Skok. [41]	Unilateral jump	Countermovement jump	√
		Lateral jump	
		Horizontal jump	
	Change of Direction	10 m shuttle	√
		20 m shuttle	
		25 m shuttle	
	Speed	10 m	√
		20 m	
		25 m	

Javier Nunez et al. [42]	Change of Direction	5 m–90º–5 m	
		5 m shuttle	√

## RESULTS

### Search results

The initial search resulted in 6365 records. After excluding 2791 duplicates, 3574 studies were selected to be screened by title and abstract. When applying the inclusion criteria, 3498 papers were subsequently excluded. The remaining 76 articles were read in full, with 62 rejected because they were acute studies (n = 21), not including both unilateral and bilateral training groups (n = 19), reviews (n = 10), participants with an age outside the criteria boundaries (n = 5), not English (n = 3), not peer-reviewed (n = 3), multiple publication (n = 1). This left 14 studies to be included in the final analysis.

### Risk of bias assessment

In accordance with the modified scale [34, 35], the scores of 14 included articles ranged from 12 to 18, the mean score was 15. Therefore, the overall quality was moderate. The majority of the studies were rated as moderate (n = 9 studies [24, 27, 29–32, 40, 42, 43]), and 5 studies were rated as good [25, 26, 28, 41, 44]. 13 studies had allocated the participants in a random manner. Most of the studies got high score in item 4 and 7, but only 28.6% of the studies used a control group. 78.6% of the studies did not clearly describe the inclusion criteria and training experience of the participants (Table 3).

**TABLE 3:** The results of risk of bias assessment

Studies	Items										
	1	2	3	4	5	6	7	8	9	10	Score
Bogdanis et al.[27]	1	2	2	2	0	0	2	2	2	0	13
Appleby et al.[26]	1	1	2	2	2	2	2	2	2	2	18
Gonzalo-Skok et al.[24]	1	2	1	2	0	0	2	0	2	2	12
Speirs et al.[28]	2	2	2	2	2	0	2	2	2	2	18
Botton et al.[29]	1	0	2	2	0	2	2	1	2	2	14
Fisher et al.[32]	1	2	2	2	1	0	2	0	2	2	14
Makaruk et al.[30]	1	2	2	2	2	2	2	0	2	0	15
McCurdy et al.[31]	1	2	2	2	2	0	2	2	2	0	15
Taniguchi et al.[43]	1	0	0	2	1	2	2	1	2	1	13
Ramirez-Campillo et al.[40]	1	2	2	2	1	0	2	1	2	2	15
Stern et al.[25]	1	2	2	2	2	0	2	1	2	2	16
Krajewski et al.[44]	2	2	2	2	2	0	2	1	2	2	17
Javier Nunez et al.[42]	1	1	0	1	2	0	2	2	2	1	12
Gonzalo-Skok et al.[41]	2	2	2	2	2	0	2	2	2	2	18

### Studies’ Characteristics

The total number of subjects was 392 (Table 4) and the participants’ age ranged from 16 to 26 years. Nine studies solely included males [24–26, 28, 32, 40–42, 44]. Three studies included both males and females [27, 31, 43], and 2 studies included females only [29, 30]. All male athletes [24–26, 28, 32, 40, 41] were from team sports, including soccer [25, 32, 40, 41], basketball [24, 26] and rugby [28]. Twelve studies had training frequency of twice per week [24–32, 40–42], and two studies had performed training 3 times per week [43, 44]. The training duration ranged from 5 to 12 weeks.

**TABLE 4:** The characteristics of the included studies

Study	Participants						Training Program						Outcome Measure
	Identity	Training Experience (years)	Age (years)	Gender	N	Frequency (times/week)	Weeks	Sets	Reps	Intensity (%1RM)	Unilateral Exercises	Bilateral exercises	Performance test
Bogdanis et al.[27]	College students	None	19.8 ± 2.9	F/M	15	2	6	2–3 3–4	10 3–8	60–90%	6 Jumps U KE U KF U	6 Jumps B KE B KF B	CMJ U CMJ B Iso-LP B MP Iso-LP U MP DJ B

Gonzalo-Skok et al.[24]	Basketball Players	> 2	16.9 ± 2.1	M	22	2	6	3	> 10% MP	80–100% MP	Bul squat	Back squat	Back squat MP Bul Squat MP CMJ B 25 m Sprint V test 15 m shuttle

Botton et al.[29]	Healthy active females	None	24.8 ± 1.4	F	43	2	12	2–4	5–15	5–15 RM	KE U	KE B	KE B 1RM KE U 1RM Iso-KE B MP Iso-KE U MP

Makaruk et al.[30]	College students	None	20.6 ± 1.3	M	54	2	12	2–8	4–15		5 Jumps U	5 Jumps B	10 sWingate SJ U SJ B

Ramirez-Campillo et al.[40]	Soccer Players	> 2	17.6 ± 0.5	M	18	2	8	1–2 3	3–5 10	70%	4 Jumps U KE U KF B	4 jumps B KE B KF B	KE B 1RM KF B 1RM T test CMJ B CMJ U SJ B SJ U H3J HCMJ H3MJ

Fisher et al. [32]	Soccer Players	> 2	19.8 ± 1.5	M	20	2	6	1–3 3	6–10 6	80%	5 Jumps U Bul squat	5 Jumps B Back squat	T test Illinois test 10 m sprint

Appleby et al.[26]	Basketball Players	> 2	22.4 ± 4.1	M	33	2	6	6–8	4–8	45–85%	Step up	Back squat	Back squat 1RM Step up 1RM 20 m sprint

Speirs et al.[28]	Rugby Players	> 1	18.1 ± 0.5	M	18	2	5	4	3–6	> 75%	Bul squat	Back squat	Bul squat 1RM 40 m sprint Pro test

Krajewski et al.[44]	Healthy active males	no	26.4 ± 5.5	M	15	3	4	3	4–6	60–90%	Bul squat Deadlift U CMJ U	Back squat Deadlift CMJ	Back squat 1RM Bul squat 1RM

Javier Nunez et al.[42]	Healthy active males	-	22.8 ± 2.9	M	27	2	6	4	7	0.05–0.1 kg/m^2^	ECC lunge	ECC Squat	CMJ 5 m shuttle 5 m 90º 5 m 10 m sprint

Gonzalo-Skok et al.[41]	Football Players	1–3	20.5 ± 2	M	48	2	8	6	6–10	0.27 kg/m^2^	6 ECC Lunges	ECC squat	CMJ CMJ U HCMJ U Lateral jump U 25 m sprint 20 m sprint 10 m sprint 10 m shuttle 20 m shuttle 25 m shuttle

Taniguchi et al.[43]	College students	None	20 ± 1.1	F/M	18	3	6	3	6		KE Isok U	KE Isok B	Isok-KE U Isok-KE B

Stern et al.[25]	Soccer Players	> 2	17.6 ± 1.2	M	23	2	6	4 4	6 3–6	75–85%	Bul squat 3 Jumps U	Back squat 3 Jumps B	Back squat 1RM CMJ B CMJ U Broad jump B Broad jump U Drop jump B Drop jump U 10 m sprint 30 m sprint 505

McCurdy et al.[31]	College students	None	20.7 ± 2.6	F/M	38	2	8	3 3–6	5–15 5–15	50–87%	2 Jumps U Bul squat	2 Jumps B Back squat	Back squat 1RM Bul squat 1RM CMJ B CMJ U

F: female; M: male; U: unilateral; B: bilateral; KE: knee extension; KF: knee flexion; Bul squat: Bulgarian split squat; Iso: isometric; Isok: isokinetic; LP: leg press; CMJ: countermovement jump; MP: maximum power; DJ: drop jump; SJ: squat jump; H3J**:** horizontal 3 jumps; HCMJ: horizontal countermovement jump; ECC: eccentric

The training modalities can be categorized into resistance training and jump training. Among them, 8 studies purely utilized resistance training as the intervention modality [24, 26, 28, 29, 41–44], 1 study purely utilized jump training [30], 5 studies combined resistance with jumping training exercises together [25, 27, 31, 32, 40]. 3 kinds of muscle actions were applied among the included studies, 11 studies used concentric overload training; 2 studies used eccentric overload training [41, 42]; 1 study used isokinetic training [43].

The unilateral multi-joint resistance exercises included step up [26, 31], Bulgarian split squat [24, 25, 28, 31, 32], single leg deadlift [44], lunge and eccentric lunge [31, 41, 42]. Unilateral single-joint resistance exercises included single knee extension and flexion [27, 29, 40, 43]. Bilateral multi-joint resistance exercises included back squat [24–26, 28], deadlift [44], eccentric squat [41, 42] and front squat [31]. Single-joint exercises included knee extension and flexion [27, 29, 40, 43]. Jump exercises included squat jump, broad jump, countermovement jump and different variants of box jumps.

The intensity of the resistance training ranged from 45–90% 1RM, and the volume, which was equal in both groups, ranged from 3 to 15 repetitions. The intensity of jumps was maximum without external loading, with repetitions and sets ranging from 3 to 15 and 1 to 8, respectively.

The outcome indicators of the 14 included studies were various. The strength-related tests included: 1) 1RM test of back squat [25, 26, 28, 31, 44], Bulgarian split squat [25, 28, 31, 44], step up [26], knee extension [29, 40], knee flexion [40] and leg press [27]; 2) maximal power test of 10 s Wingate [30], Margariakalamen stair-climb test [31], isokinetic knee extension [43], back squat and Bulgarian split squat [24]. The jump-related tests (unilateral and bilateral) included squat jump and countermovement jump [24, 27, 30, 31, 40–42], broad jump [25, 41], drop jump [25, 27, 40], 3 steps and 5 steps jump [30, 40]. The COD-related tests included the 50° test [26], the V test [24], 180° shuttle [24, 41, 42], the pro-agility test [28], the T test [32, 40], the 505 test [25] and the Illinois test [32]. The speed-related tests included 10 m sprint running [25, 28, 32, 41], 20 m sprint running [26, 41], 25 m [24, 41] and 40 m sprint running [25, 28]. After dealing with the multiplicity, the final included outcomes for analysis were showed in table 2.

### Quantitative analysis

**FIG. 2:**
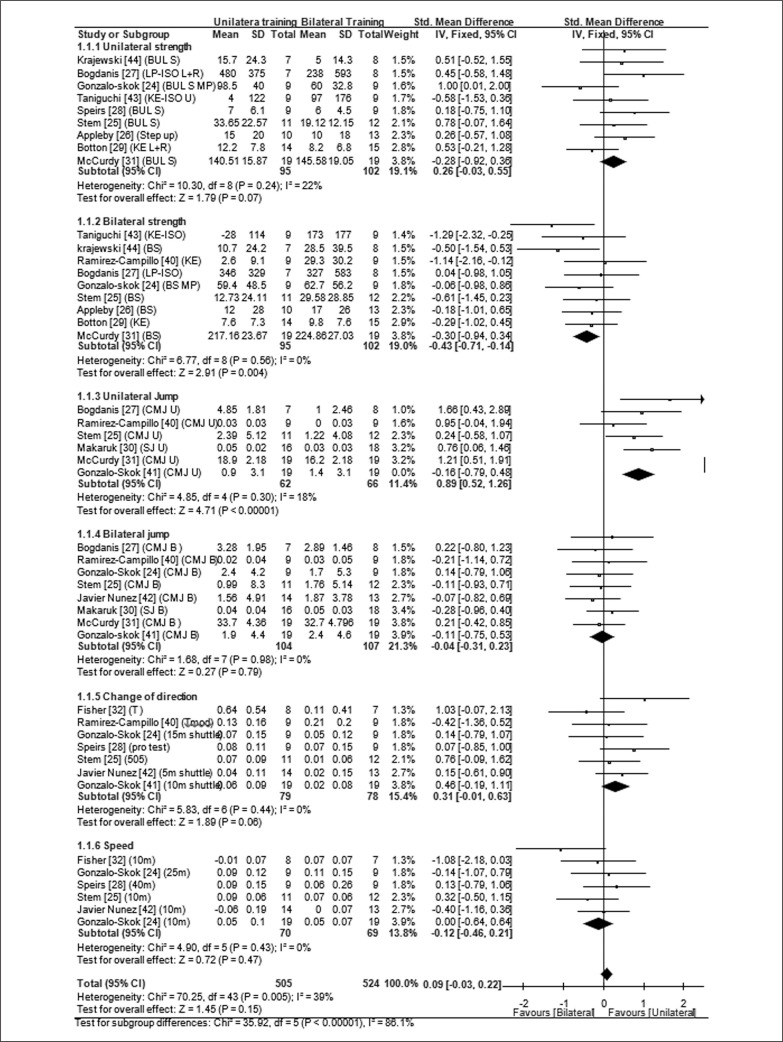
Forest plot comparing the effects of bilateral and unilateral exercises training on athletic performance. Bul s: Bulgaria split squat; LP: leg press; ISO:isokinetic; MP:Maximum power; KE: knee extension; U: unilateral; B:bilateral; L: left; R: right; CMJ: countermovement jump; SJ: squat jump; Tmod: T modified test.

The performance tests in included studies can be categorized into 6 subgroups, namely: unilateral strength, bilateral strength, unilateral jump, bilateral jump, COD and speed performance tests. After sensitive analysis, the I^2^ < 50% in all subgroups suggested a non-significant heterogeneity, and the fixed effects models were used to aggregate the SMDs. The total heterogeneity (Chi^2^ = 70.25, *p* = 0.03; I^2^ = 39%) of 44 effects was medium. The overall effect size was non-significant and classified as trivial effect (ES = 0.09; *p* = 0.15; 95% CI: -0.03, 0.22) (Figure 2). The heterogeneity for inter subgroup differences was large (I^2^ = 86.1%).

### Unilateral strength performance

After dealing with the multiplicity (Table 2), 9 outcomes were selected for meta-analysis. The heterogeneity was low (Chi^2^ = 10.30, *p* = 0.24; I^2^ = 22%). In fixed effects model, overall effect was not significant (*p* = 0.07). Pooled effect size and 95% CI were 0.26 (-0.03, 0.55) and classified as a small effect.

### Bilateral strength performance

After dealing with the multiplicity, 9 outcomes were selected to do meta-analysis. The heterogeneity test was not statistically significant (Chi^2^ = 6.77, *p* = 0.56; I^2^ = 0%). In fixed effects model, overall effect was significant (*p* = 0.004). Pooled effect size and 95%CI were -0.43 (-0.71, -0.14) and classified as a small effect.

### Unilateral jump performance

After dealing with the multiplicity, 6 outcomes were selected for meta-analysis. The original heterogeneity was high (I^2^ = 60%), after alternated sensitive analysis, one study with eccentric overload training different from others was excluded, and the adjusted heterogeneity was low (Chi^2^ = 4.85, *p* = 0.30, I^2^ = 18%). In fixed effects model, the pooled effect size and 95% CI was 0.89 (0.52, 1.26) with statistical significance (*p* < 0.0001) and classified as a large effect.

### Bilateral jump performance

After dealing with the multiplicity, 8 outcomes were selected for meta-analysis. The heterogeneity was not statistically significant (Chi^2^ = 1.68, *p* = 0.98; I^2^ = 0%), in fixed effects model, the pooled effect size and 95%CI was -0.12 (-0.40, 0.15) with non-significance (*p* = 0.79) and classified as a trivial effect.

### COD performance

After dealing with the multiplicity, 7 outcomes were selected for meta-analysis. The heterogeneity was not statistically significant (Chi^2^ = 5.83, *p* = 0.44; I^2^ = 0%). The pooled effect size and 95%CI was 0.31 (-0.01, 0.63) with non-significance (*p* = 0.06) and classified as a small effect.

### Speed performance

After dealing with the multiplicity, 6 outcomes were selected for meta-analysis. The heterogeneity was not statistically significant (Chi^2^ = 2.38, *p* = 0.67; I^2^ = 0%). in fixed effects model, the pooled effect size and 95%CI was -0.12 (-0.46, 0.21) with non-significance (*p* = 0.82) and classified as a trivial effect.

## DISCUSSION

This systematic review with meta-analysis aimed to compare the training effects of unilateral vs. bilateral resistance training on measures of athletic performance. The results showed that: 1) both unilateral and bilateral resistance training improve athletic performance; 2) with respect to all performance measures, overall effect showed no difference between unilateral resistance training and bilateral resistance training; 3) based on the subgroup analysis, the unilateral resistance training showed a large effect on improving unilateral jumping performance, but non-significant difference on improving unilateral strength performance in comparison to bilateral exercises. In contrast, bilateral resistance training exhibited a small effect on improving bilateral strength performance, but non-significant differences on improving bilateral jumping performance in comparison to unilateral training. Finally, COD and speed performance improvement showed no differences between unilateral and bilateral resistance training; however COD, which involves unilateral propuslive force production, did show a small effect in favor of unilateral resistance training.

### Strength performance

The outcome of bilateral strength performance subgroup supported our hypothesis that bilateral resistance training followed the training principle of specificity, which demonstrated a small effect (ES = -0.43) on improving bilateral strength performance in comparison with unilateral resistance training. It is clear that the muscle cross-sectional area and neuromuscular adaptation are the main factors for developing maximum strength [45]. There is evidence that unilateral and bilateral strength traning had a similar impact on muscle mass [46], girth [29] and cross-sectional area [47]. However, Helme et al., [48] found that almost 15% of the load was placed on the rear leg during the Bulgarian split squat. It could be inferred that the rear leg might be contributed to the concentric phase, which means that the lead leg might decrease force development. Anderson et al., [49] and McCurdy et al [50] found that the bilateral squat activated the kneejoint agonists (e.g., quadriceps) greater than the Bulgarian split squat, while the Bulgarian split squat showed greater antagonists such as the hamstrings, hip abductors and trunk musculature. Cumulatively, it could be speculated that bilateral strength training may produce greater knee agonist neuromuscular adaptation owing to higher load other than the muscle growth.

Four studies in this systematic review showed that the bilateral group mitigated the bilateral force deficit (BLD), but unilateral resistance training increased the BLD in measures of knee extension, isometric leg press and squat with one leg and both legs, respectively [24, 27, 29, 43]. BLD is described as the sum of the maximum forces exerted by the left and right limbs unilaterally as being greater than the simultaneous exertion of both limbs bilaterally [51]. More recently, Bishop et al. found that a combination of bilateral and unilateral resistance training had superior effects on unilateral jump performance compared to bilateral. Consequently, it stands to reason that if greater improvements in unilateral jump performance are evident (compared to bilateral), it will have an effect on the BLD outcome (remembering that the BLD is a product of both unilateral and bilateral scores, presented as a single ratio number) [52]. In line with the definition and the findings by Bishop et al., the results of these 4 studies might be explained by the reason of specificity. Simply put, that unilateral resistance training is likely to enhance unilateral performance measures more than bilateral performance measures, and vice versa. Given the inherent differences in study design of these 4 studies (e.g.different levels of stability requirements in the chosen methods but still with similar effects on the BLD), it is speculated that neuromuscular factors induced by the mechanism of BLD may be more of a contributing factor to changes than stability.

The results of unilateral strength performance subgroup indicated that unilateral resistance training had a small effect (ES = 0.26) on improving unilateral strength. However, there were not statistically significant between-group differences. Our finding did not correspond well with included studies which found clear evidence that unilateral resistance training was better for improving unilateral strength performance [24, 26]. The possible explanation was that these studies did not adjust each intervention group’s mean changes between pre and post tests for analysis as in the current study, which may enlarge the probability to make the false inference due to the selection effect. For example, McCurdy et al., [31] found that 8 weeks’ unilateral resistance training was more effective in improving 1RM of a Bulgarian split squat than the bilateral resistance training. However, after adjusting the pretest difference, both groups exhibited similar effects on the 1RM of Bulgarian split squat. In addition, the unilateral exercises may stimulate the stabilizing muscles in the core and knee to a greater extent than bilateral exercises [50, 53], which are likely to be beneficial for improving stability and force transference through the kinetic chain. However, the greater agonist neuromuscular adapation of bilateral resistance training may counteract the superiority of stability and specificity of unilateral exercises in unilateral strength tests. Therefore, it is up to coaches to determine what the athlete needs and program accordingly.

### Jump performance

The larger improvement in unilateral jump performance induced by unilateral rather than bilateral resistance training corresponded with the training principle of specificity. Five studies were included in the unilateral jump subgroups, all of which applied jumping exercises as intervention modalities [25, 27, 30, 31, 40] and found that the effect of unilateral jumping training were better (ES: 0.24–1.66) than bilateral jumping training. These findings might result from the differences in stability and neuromuscular adaptation of agonists. In unilateral jumping, the smaller supporting surface triggers a higher co-activation in the stabilizing muscle groups (e.g. hamstrings), and helps maintain the head, arms, trunk and lower limbs in the same direction during both landing and taking off. As a result, the stability of the lower limbs are improved, and the absorption of the reaction force of the lower limbs decreases during the landing phase [54]. Bogdanis et al. [27] found that unilateral jumping training was more effective at improving unilateral squat jump performance and RFD_0–50 ms_ and RFD_0–100 ms_ (Rate of force development) in unilateral isometric seated leg extension in comparison to bilateral jump training. Turki et al. [55] also found that the electromyographic level of the vastus intermedius and gastrocnemius in unilateral body weight vertical jump was 10–25% higher than that in bilateral body weight vertical jumps. According to the force-velocity relationship, unilateral jumping without a second limb to ‘spread the load’ needs to push a greater relative load with a longer time to take off. It could be inferred that unilateral jumping might make the muscles generate more force, and more strongly stimulate the muscles such as the extensors of the ankle, knee and hip to result in greater neuromuscular adaptation when considering individual limbs. It should be noted that 1 study not included for aggregating effect size after sensitive analysis purely used eccentric overload resistance exercises as intervention modalities and found bilateral training group improved unilateral countermovment jump better than the unilateral exercise group [41]. Although speculative, the results may be attributed to the eccentric force improvement in bilateral group which help to stablize the posture during testing. In addition, the training status of subjects will also have an effect on results. For example, it is plausible that high-level athletes might exhibit less improvements in bilateral jump performance (compared to non-athletes), as there is arguably a reduced ‘window of opportunity’ for enahnced adapatation [56]. However, it could be suggested that improvements in unilateral jump performance may be more likely for all, owing to the use of unilateral resistance training methods being employed less frequently. This concept is partly supported by the findings of Bishop et al. [53] who showed sigificant improvements in unilateral jump height (but not bilateral), after an 8-week combined bilateral and unilateral strength training programme. Collectively, these evidences may explain why unilateral resistance training had a larger effect on improving unilateral jumping performance in comparison with bilateral resistance training.

The overall effect size of bilateral jumping subgroup was trivial in favor of bilateral exercises training, but the value was not statistically significant. The results may be the contribution of the mechanism of bilateral deficit and differences in kinetics [57]. When both legs contract simultaneously, the nerve activation of the left and right interhemisphere may be mutually inhibited, and there was a lack of bilateral activation while training unilaterally, so it would be expected that muscles would not achieve maximum voluntary contraction in unilateral jump group. Furthermore, the contact time for bilateral and unilateral jumps (CMJ) was 178–190 ms and > 250 ms respectively [21]. As above explained, the unilateral jump needs to push relative higher load, which may result in greater range of motion and deeper squat depth for producing greater force, subsequently increase the contact time. Thus, it is conceivable that the unilateral jump group might be unable to adapt in the shorter exertion time to develop similar force as the bilateral jump group in the measure of bilateral jump test [58]. In contrast, bilateral jump exercises similar with the test modality may have a priority of familiarization and be adapted to exert force in shorter time.

### Change of direction (COD) and speed performance

COD speed and sprint have been suggested to be largely unilateral exercises in previous research [12, 59]. However, our results indicated that the effect size in the speed subgroup showed no difference between unilateral and bialteral resistance training, similar to recent findings [33]. However, COD was likely to be in favor of unilateral resistance training with a small effect.

Four of the 7 studies in the COD subgroup that adopted both the Bulgarian split squat versus back squat as the modalities of resistance intervention [24, 25, 28, 32] supported use of the Bulgarian split squat over the back squat. McCurdy et al. found that the Bulgarian split squat could produce greater activation in glutes maximus and hamstring compared with back squat [50]. Another 2 studies in this subgroup used eccentric overload as the intervention modality also supported the unilateral group as being the more favourable method on enhancing COD performance. Chaabene et al., [60] obtained a *moderate* to *very large* correlation (*r* = 0.45–0.89) between eccentric strength and the COD performance through a systematic review. The concentric force of push, the eccentric force during decelerating, and the ground contact time are the key factors affecting the ability to COD effectively [61, 62]. A larger eccentric braking force can shorten the deceleration time, increase the elastic force of the muscles and connective tissue, and generate a greater subsequent push force, which collectively results in a shorter ground contact time, greater acceleration, and thereby improving overall COD performance [62]. Accordingly, the unilateral exercises such as Bulgarian split squat emphasizes the use of eccentric force in a predominantly unilateral manner, which could explain the greater improvement on COD performance.

The current data also demonstrated that bilateral resistance training had a trivial effect on improving linear speed performance. Various factors account for the improvement of speed performance. Empirical evidence shows that the rate of force development in short time and large propulsive forces in initial acceration stage are the main attributes of speed performance. Wilkau et al. [63] found that the contact time, vertical force and peak propulsive forces (r = –0.64, r = 0.57 and r = 0.66, respectively) contributed most to the step velocity. In a meta analysis of 15 studies, Seitz et al. [64] reported a strong relationship between squat strength and sprint performance (*r* = -0.77; *p* = 0.001). In the current study, the strength subgroup showed that bilateral exercise training had a larger effect in improving bilateral strength with statistical significance. Collectively, the improvement of bilateral strength may explain the trivial effect in favor of bilateral exercises training on improving speed.

It should be noted that the overall effect of each training method (unilateral vs bilateral) on COD and speed were not statistically significant. This could be attributed to the studies’ small sample size, the lack of specificity of training methods with regards to the tests measures, and/or the low transference of jump and resistance training adaptations to sprint and COD performance measures [65]. However, Hopkins et al. [66] stated the smallest worthwhile enhancement (SWE) by 10% will help the athletes to win the game. SWE was calculated by the coefficient of the variablility (CV) of withinathlete’s performance from competition to competition. With respect to short running events, it was found SWE was even lower to 0.3–0.5% [67]. This indicated that although the effect sizes were rather small or not significance between unilateral vs. bilateral resistance training, when inter-athlete variability was counted, the small difference in improving performance caused by the exercise selection were within a range that was meaningful for elite athletes. Thus, these findings still have significant implications for understanding how to choose unilateral or bilateral resistance exercise for optimizing speed performance.

It should be acknowleged that some limitations exist in our paper. Firstly, the lower number (< 10) of studies in subgroup analyses means it was not possible to do further meta-regression analysis. Thus, as is often the case in science, more research is likely needed to provide greater clarity between the two training methods. Secondly, all included studies for this meta-analysis applied lower body exercises as training intervention, thus, whether the present findings based on lower limbs studies can be applied to the upper limbs remains unclear. Future research should attempt to investigate the difference between bilateral and unilateral exercises training for the upper limbs. Thirdly, the included participants’ age ranged 16 to 26 years, which means that our results may not be extrapolated to those participants with age out of this range. But it still can provide references for these practitioners.

## CONCLUSIONS

This study set out to establish the different effects between unilateral and bilateral resistance training on strength, jump, linear and COD speed performance. The most interesting findings was that unilateral resistance training was superior for enhancing unilateral jump performance, and bilateral resistance training was superior for enhancing bilateral strength performance. But both had no significant difference in enhancing unilateral strength, bilateral jump, linear and COD speed performance. The results suggest that both kinds of training should likely be considered by practitioners. Specifically, bilateral exercises should be chosen for enhancing bilateral strength performance, and unilateral exercises should be chosen for enhancing unilateral jump performance. With regards to other performance outcomes (e.g., linear and COD speed), practitioners can probably choose any kind of exercises based on their interest and the availability of facilities.

## Funding

This work was supported by the Winter Olympics Foundation [2018FF0300901] and China Institute of Sport Science Basic Foundation [Basic 17–30] grants to Yong-ming Li.

## Conflict of interest

The authors declare that they have no competing interests.
